# Structural and Mechanical Dynamics of Polymer Membranes Across Multilength Scales

**DOI:** 10.1002/advs.202521391

**Published:** 2026-01-20

**Authors:** Rifan Hardian, Hakkim Vovusha, Yue Yuan, Changxia Shi, Eugene Y.‐X. Chen, Mario Lanza, Gyorgy Szekely

**Affiliations:** ^1^ Advanced Membranes and Porous Materials Center, Physical Science and Engineering Division King Abdullah University of Science and Technology (KAUST) Thuwal Saudi Arabia; ^2^ Chemical Engineering Program, Physical Science and Engineering Division King Abdullah University of Science and Technology (KAUST) Thuwal Saudi Arabia; ^3^ Physical Science and Engineering Division King Abdullah University of Science and Technology (KAUST) Thuwal Saudi Arabia; ^4^ Department of Chemistry Colorado State University Fort Collins CO USA; ^5^ Department of Materials Science and Engineering, 9 Engineering Drive 1 National University of Singapore Singapore Singapore; ^6^ Institute For Functional Intelligent Materials National University of Singapore Singapore Singapore; ^7^ Centre For Advanced 2D Materials (CA2DM) National University of Singapore Singapore Singapore

**Keywords:** AFM nanomechanics, in situ SAXS–WAXS, mechanical heterogeneity, polymer membrane, surface and bulk properties

## Abstract

Understanding the mechanical and structural evolution of polymer membranes under heat and strain is important for many applications. Conventional techniques, such as dynamic mechanical analysis provide bulk mechanical information but lack the spatial resolution to capture localized variations. Similarly, X‐ray diffraction spectroscopy effectively probes long‐range order but has limited capability in analyzing amorphous polymer structures. Herein, we reveal the importance of mechanical and structural analyses across multilength scales. We unveiled the opposite trend in surface‐to‐bulk mechanical behavior of polymer membranes, necessitating the investigation of both regions to fully capture their functional behavior. We mapped nanoscale mechanical inhomogeneities across membrane surfaces with in situ atomic force microscopy quantitative nanomechanics. Further, we uncovered structural irregularities across both short‐ and long‐range order using in situ small‐ and wide‐angle scattering spectroscopies. We investigate key structural parameters and describe density variations in amorphous domains. Molecular dynamics simulations corroborate with the observed structural and mechanical properties at the molecular level. Our multilength‐scale characterization strategy provides a robust framework for elucidating structure–property relationships from macroscopic to molecular levels. The approach is generalizable to other systems such as films, fibers, and two‐dimensional materials, enabling new insights into their dynamic properties.

## Graphical abstract

Distinct surface‐to‐bulk mechanical contrasts in polymer membranes were unveiled, highlighting the need to probe both regions to fully capture their functional behavior. By integrating in situ SAXS–WAXS with AFM‐based nanomechanics, nanodomain‐level heterogeneity and real‐time structural transformations induced by thermal and mechanical stimuli were revealed, offering a deeper understanding on the structural and mechanical behavior of the membranes.

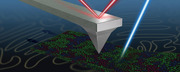

## 1 Introduction

As semi‐permeable filter media, polymer membranes play essential roles in industrial separation processes such as water purification, gas separation, pharmaceutical preparation, food packaging preparation, and protein purification [1]. The fundamental understanding of polymer membrane properties relies heavily on the material characterization techniques. As the properties of polymer membranes at local scale may differ from their bulk properties, appropriate characterization techniques are required to reveal information from both local and bulk states. For example, Cavalcante et al. reported an interpenetrated polymer network membrane, which apparently showed isotropic chemical properties in the bulk when analyzed using infrared spectroscopy analyses; however, local nanoinfrared analyses revealed inhomogeneities in the chemical properties [2]. Furthermore, chemical properties of the polymer membrane at its surface can also differ from its cross‐section [3]. In addition to the variation in chemistry, the mechanical properties of the membranes may also vary at the local scale [4, 5, 6]. Therefore, characterization techniques must be complemented with local and bulk analyses to comprehensively understand the properties of polymer membranes.

The bulk mechanical properties of membranes are conventionally determined at the centimeter‐scale using techniques such as dynamic mechanical analysis (DMA) [2, 7, 8], However, DMA cannot reveal local‐scale mechanical properties, such as the stiffness distribution of the membrane surface and mechanical inhomogeneity within a sample. Nanoindentation provides nanoscale mechanical information but cannot map the distribution of the mechanical properties throughout the sample. To reveal the nanoscale mechanical information, atomic force microscopy (AFM) PeakForce quantitative nanomechanics technique has been employed [4, 9, 10]. This technique enables the direct visualization of the mechanical properties including modulus, adhesion, dissipation, and deformation, at high spatial resolution, determined by the radius of the AFM tip apex (2–25 nm).

**FIGURE 1:**
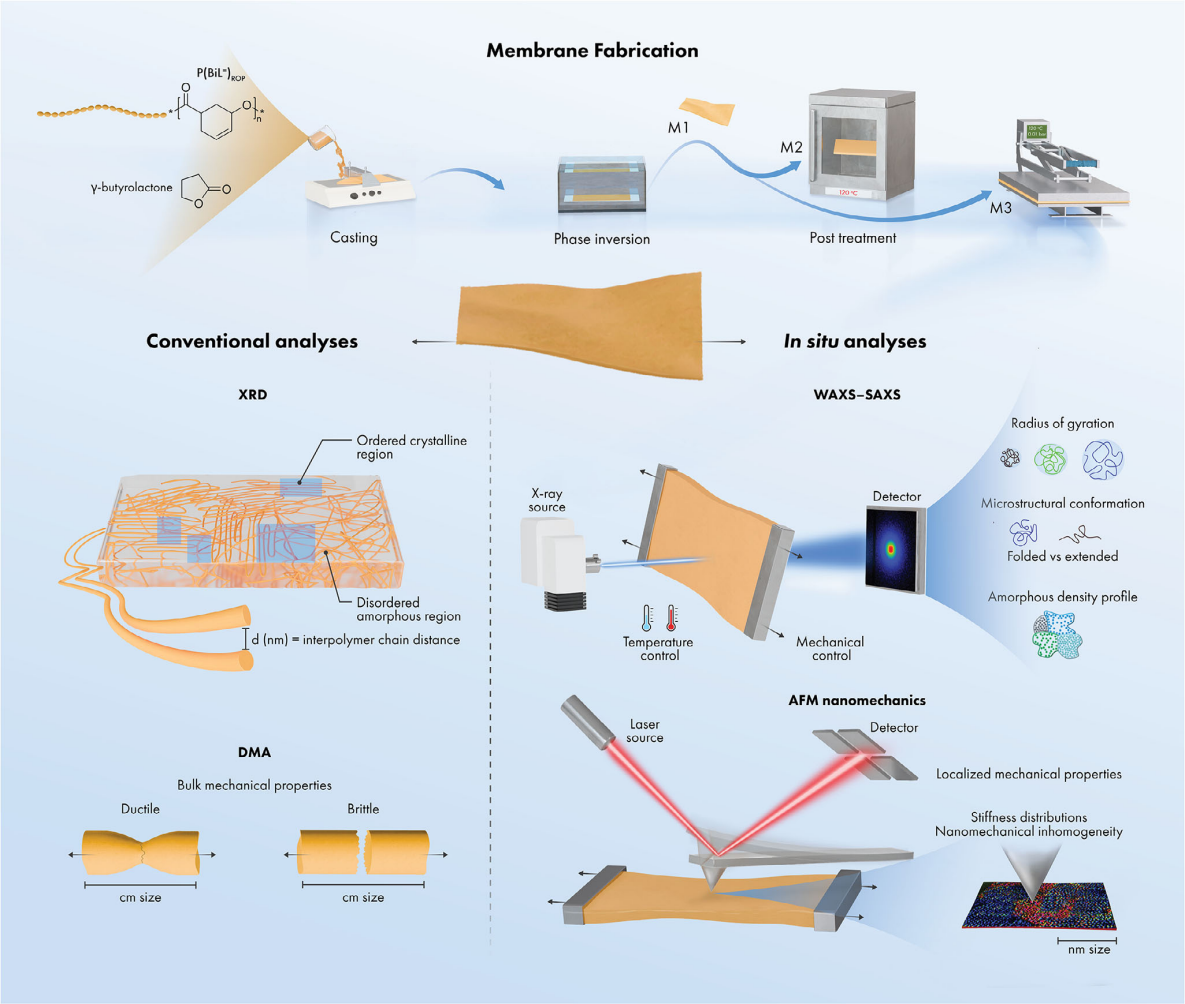
Mechanical and structural information obtained from various techniques. Membrane fabrication involves casting polymer dope solution followed by phase inversion and post treatments; M1 was prepared by drying the membrane at room temperature, M2 was prepared by annealing the membrane at 120°C for 2 h, and M3 was prepared by hot‐pressing at 120°C and 0.01 bar for 2 h. Conventional DMA and XRD provide limited mechanical and structural information, which could be extended by in situ WAXS, SAXS and AFM nanomechanic techniques. In situ techniques with temperature and mechanical controls enable the investigation of structural and mechanical dynamics under the influences of heat and tensile forces.

X‐ray diffraction (XRD) and wide‐angle x‐ray scattering (WAXS) techniques have successfully unveiled the long‐range ordering and crystallinity in polymer membranes [11], although XRD provides limited information on amorphous polymer membrane systems. Supramolecular assemblies in amorphous systems give rise to density fluctuations, which typically occur at the dimension scale (10–1000 Å) that far exceeds the analytical limit of XRD [12]. Alternative structural analysis techniques such as small‐angle X‐ray scattering (SAXS) are required for disclosing the structural information of amorphous materials [13, 14]. The comparison of conventional DMA and XRD techniques with advanced in situ AFM and SAXS techniques are illustrated in Figure 1.

Polymer membranes used in practical applications such as those related to filtration [15], fuel cells [16], piezoelectric fabrication [17], and miniaturized creep testing [18] should withstand mechanical stretching. In particular, membranes used in spiral‐wound membrane modules must withstand the mechanical force of rolling. Microporous membranes are often stretched during fabrication to modulate their porosity [19, 20]. Stretching influences not only the morphological properties but also the mechanical and structural properties of membranes. Membrane behavior can be comprehensively understood by investigating their structural and mechanical properties under a tensile force. Insight into mechanical responses at the nanoscale under applied strain is essential, as key membrane performance parameters—including permeability, selectivity, resistance to fouling, and long‐term stability—are strongly governed by the behavior of the polymer network at these length scales. In this study, we investigated the structural and mechanical properties of polymer membranes at the bulk and local scales using a combination of in situ techniques. By installing stretching platforms on the SAXS/WAXS and AFM instruments, we acquired the structural and mechanical information of polymer membranes under a uniaxial tensile force.

Annealing, which relieves internal stresses during membrane fabrication, is widely adopted for improving the gas permeation properties [21], modulating the proton conductivity [22], and enhancing the chemical resistance via thermal crosslinking [23] of polymer membranes. Therefore, by investigating the structural and mechanical evolution of polymer membranes during heat treatment, we can reveal their material characteristics in real applications. In this work, we investigated the structural dynamics of polymer membranes subjected to various temperature treatments using in situ temperature‐programmed SAXS/WAXS.

In situ SAXS/WAXS and AFM quantitative nanomechanic techniques can analyze diverse materials, including free‐standing membranes, membrane supports, mixed‐matrix membranes, films, 2D materials, and fibers. Our work demonstrated the applicability of these techniques to free‐standing membranes fabricated from the P(BiL ^=^)_ROP_ polymer, which was recently classified as a circular polymer that exhibits a sustainable end‐of‐life cycle [24]. The membranes were fabricated using a green solvent and demonstrated separation performance in nanofiltration [7]. To investigate the effects of morphology on the mechanical and structural dynamics of the membranes during stretching, we modified the membranes through annealing and hot‐pressing. Annealing is known to modulate the mechanical properties and crystallinity of membranes [25, 26], and hot‐pressing simultaneously modulates the morphology and thickness of membranes, influencing the structural and mechanical properties [7].

In situ SAXS/WAXS and AFM nanomechanics analyses reveal the local and molecular‐level insights of polymer membranes, overcoming the limitations of conventional techniques such as XRD and DMA. In this study, we investigated density variations in amorphous domain as well as the description of the amorphous boundary, structural conformation, stiffness distribution, and mechanical inhomogeneity on membrane surfaces. The experimentally observed effects of heat and mechanical treatments on the chain flexibility and fractional free volume (*FFV*) of polymers were corroborated by molecular dynamics (MD) simulations. To characterize bulk properties of the polymer membranes, we employed a combination of characterization techniques, including DMA, DSC, TGA, and SEM. By integrating characterization techniques across multiple length scales (from macroscopic to molecular) with MD simulations, we comprehensively elucidated the structure–property relationship of polymer membranes.

## 2 Experimental Section

### 2.1 Membrane Fabrication

To fabricate P(BiL ^=^)_ROP_ polymer membranes, 7 g of γ‐butyrolactone was added to 3 g of the P(BiL ^=^)_ROP_ polymer to obtain a 30‐wt% polymer doped solution, which was homogenized by stirring at 150 rpm and room temperature for 24 h. Further, degassing was performed under a nitrogen atmosphere in an incubator shaker at 200 rpm and 25°C for 4 h to ensure that the dope solution was free from any entrapped gas. Membranes were cast onto a glass plate using an Elcometer 4340 film applicator with a 200‐µm‐thick casting knife. The cast film was immediately immersed in a water coagulation bath at room temperature (23°C) for 24 h, yielding membrane M1. Membrane M2 was prepared via the same procedure as followed for preparing M1 but with annealing at 120°C for 2 h. Membrane M3 was prepared via the same procedure as followed for preparing M2, but annealing was replaced by hot‐pressing between two glass plates at 0.01 bar. The membrane fabrication process was shown in Figure 1.

### 2.2 Membrane Characterization

The membrane structures were derived from WAXS and SAXS measurements using a high‐resolution X‐ray scattering system (Xenocs Xeuss 3.0) with a Genix3D Cu Kα radiation source (wavelength λ = 1.542 Å, 50 kV, 60 mA) and a Dectris Eiger2 4 M detector. The measuring time of each SAXS and WAXS spectrum was 60 s. The sample‐to‐detector distances of the SAXS and WAXS measurements were 100 and 450 mm, respectively, in “Standard” collimation mode. The transmitted flux was approximately 45 Mph s^−1^. For the scattering measurements, the membrane samples were cut into pieces resembling dog bones and attached to a modular force stage (Linkam MFS350). The stretching stage was stopped at 0.5%‐strain increments for collecting the SAXS and WAXS data. For grazing incident‐WAXS (GI‐WAXS), the sample‐to‐detector distance was kept at 72 mm for GI‐WAXS. All patterns were collected with an incident angle of omega = 0.25°. The measuring time was set to 300 s. Frames were taken on a Dectris Eiger2 4 M detector, and data processing steps, including background correction (electronic noise), cosmic radiation elimination, and 2D to 1D integration, were performed afterward.

The crystallite size of polymers can be quantified using the Scherrer equation:

(1) $$D=\frac{k\lambda }{\beta cos\theta }\;$$

where *D* was the crystallite size (nm), *k* was the shape factor (0.95–0.98), *λ* was the Cu K_α_ wavelength (0.154 nm), *β* was the full width at half maximum, and *θ* was the diffraction angle, which can be correlated to the scattering vector *q* through the modified Bragg's equation:
(2) $$\lambda =2\left(\frac{2\pi }{q}\right)sin\theta$$

The Guinier plot was useful to estimate the radius of gyration, which measures the average distance of the monomers in the polymer chain within the globule from their common mass center.

(3) $$\ln I=\ln I_{0}-\frac{1}{3}q^{2}{R_{g}}^{2}$$

where I_0_ was the scattered intensity at q = 0 (at zero angle) and I was the scattered intensity at the reciprocal parameter q, and Rg was the radius of gyration. The radius of gyration can be determined from the slope of ln I against q^2^.

Kratky plots of SAXS intensity data were commonly used for qualitative assessment of polymer disorder. The degree of protein disorder can be inferred from a visual inspection of a plot of q^2^I(q) versus q. Compact proteins will have q^2^I(q) values that approach zero (or baseline) at high q, while unfolded, or disordered, proteins will generally plateau at intermediate angles followed by continuously increasing values of q^2^I(q) at wide angles. An alternate version of a Kratky analysis renders (qR_g_)^2^I(q)/I(0) versus qR_g_. The x‐ and y‐axes of these plots were dimensionless and therefore were independent of the size and molecular weight of the molecule of interest. Hence these normalized or dimensionless Kratky plots were useful for the analysis of SAXS profiles across different systems.

The Porod plot can be used to determine the density fluctuations in a multiphase system:

(4) $$\ln\left[q^{4}I\left(q\right)\right]=lnK+bq^{2}$$

where I was the scattering intensity, q was the reciprocal parameter, K was the Porod constant, and b was a constant related to the size of the regions with micro‐fluctuations of electron density.

The bulk mechanical properties of the membranes were derived from strain and stress measurements performed at room temperature under a 0.05‐N min^−1^ ramped force (DMA Q800). Differential scanning calorimetry (DSC) was performed under a nitrogen atmosphere at a heating rate of 5°C min^−1^ (TA Discovery 250). A temperature range of 300–350°C was applied, and the samples were subjected to a single heating run. TGA data were collected using a Netzsch TG 209 Iris, with a sample mass of 5 mg. The measurements were performed from 25 to 500 °C at a heating rate of 10 °C min^−^^1^ under an oxygen atmosphere. Fourier transform infrared spectroscopy (FTIR) was performed with 64 scans at 4 cm^−1^ resolution in the 600–4000 cm^−1^ wavenumber range (Nicolet iS10, Thermo Scientific).

The membranes were characterized using AFM (Bruker Dimension Icon) operated in PeakForce Quantitative Nanomechanics mode. For precise mechanical testing, the AFM system was integrated with a Deben micro tensile machine (Deben Microtest 2 kN tensile stage) (see Figure S5). This system was designed to accommodate a wide range of materials and testing conditions, with interchangeable modules and load cells ranging from 2 N to 2 kN, selected according to the sample type and required force range. A schematic illustration of the tensile stage configuration was shown in Figure S5. The tensile stage was operated using the manufacturer‐supplied acquisition software, which enables precise control of the tensile module and real‐time recording of force‐extension curves. For the measurements reported in this work, a load cell with a maximum capacity of 300 N was used. The motor speed was set to 0.2 mm min^−1^, with a gain setting of ×1, corresponding to full‐scale force display (0–300 N) in the acquisition window. The initial claw distances were 33.286 mm, 33.759 mm, and 33.089 mm for membranes M1, M2, and M3, respectively. Measurements were performed with a silicon ScanAsyst‐air tip with a spring constant of approximately 0.4 N m^−1^ and a nominal tip radius of approximately 2 nm (as specified by the manufacturer), offering the necessary sensitivity for high‐resolution characterization. The tip was calibrated on a standard sapphire substrate prior to each measurement. For the calibration, a ScanAsyst‐Air probe and a sapphire reference sample were used. Deflection sensitivity was first calibrated by scanning a 1 µm × 1 µm area at a scan rate of 1 Hz, with a peak force setpoint of 0.1 V and auto‐control enabled. The engage setpoint was 0.15 V. After identifying a clean and flat region, ramp mode was used with a trigger threshold of 0.5 V, deflection error as the data type, relative trigger mode, and a ramp size of 500 nm. Five ramp curves were acquired to obtain an averaged deflection sensitivity, which was manually entered into the calibration window. The background of the force curve was also corrected after the calibration. This calibration process was important for accurately determining the deflection sensitivity and extracting the suitable Sync distance parameters for the quantitative nanomechanics mode (see Figures S6 and S7). The spring constant was calibrated using the Thermal Tune method. Later, the Sync Distance QNM value was determined from the force‐time curve and recorded in the Supporting Information (see Figure S6). This calibrated value was used consistently for all subsequent measurements and performed individually for each tip that used. Each membrane was carefully scanned under controlled conditions to ensure minimal perturbation of the sample while capturing high‐resolution topographical and mechanical data. The total scan area was 10 × 10 µm with a resolution of 256 × 256 pixels, yielding 65,536 data points (analysis locations). From these, we selected 100 representative data points, spaced approximately 900 nm apart, for determining the adhesion force and DMT modulus. Thus, the representative data points refer to these selected data points rather than to the pixels themselves. The average values for adhesion and modulus were then calculated from these 100 representative points. An illustration of this methodology was provided in Figure S15.

### 2.3 Molecular Dynamic Simulations

Molecular dynamics (MD) simulations were performed using Materials Studio 2023 with the well‐validated Condensed‐phase Optimized Molecular Potentials for Atomistic Simulation Studies (COMPASS) force field. The constructed polymer cells were first energy‐minimized, followed by 5 ns simulations in the constant number of atoms, volume, and temperature (NVT) and constant number of atoms, pressure, and temperature (NPT) ensembles. The temperature and pressure control were maintained using the Nosé and Berendsen methods, respectively. The free volume of the polymer was characterized by the fractional free volume (*FFV*) parameter, calculated using the following equation:

(5) $$FFV=\frac{V_{f}}{V_{0}+V_{f}}$$

where *V_f_* and *V*_0_ represent the free and occupied volumes of the polymer matrix, respectively.

The compactness of the polymer chains was evaluated as the squared radius of gyration *R_g_* given by the following equation:

(6) $$R_{g}^{2}=\frac{\sum mr^{2}}{\sum m}$$

where *m* was the mass of each particle, and *r* was the distance of each atom from the center of mass.

The mean square displacement (*MSD*) of the polymer chains was computed using the following equation:

(7) $$MSD=\frac{1}{N}\sum_{i=0}^{N-1}{\left(r_{i}\left(t\right)-r_{i}\left(0\right)\right)}^{2}$$

where *r*(0) and *r*(*t*) denote the atomic positions at the initial time and time *t*, respectively, and *N* was the total number of atoms.

The stress–strain behavior of the uniaxially oriented models was evaluated using the Stress–Strain script in Materials Studio 2023, employing molecular dynamics simulations with the COMPASS II force field [27]. The spatial orientation correlation function (SOCF) quantifies the orientational correlation of two backbone chemical bonds in a polymer system, considering both intra‐molecular and inter‐molecular bond pairs using the equation:

(8) $$P_{2}\left(\cos\theta \right)=\frac{3cos^{2}\theta -1}{2}$$

where θ denotes the angle between the units. Perfect orientation corresponds to θ values of 0° or 180°, for which *P_2_* (*cos θ*) = 1. In contrast, a random orientation occurs when θ = 90°, yielding *P_2_* (*cos θ*) = 0.

## 3 Results and Discussion

### 3.1 Structural–Mechanical Relationship of the Polymer Membranes

**FIGURE 2:**
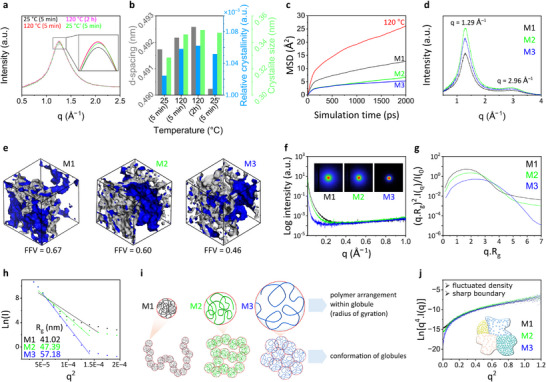
Structural analyses of the polymer membranes. Temperature‐programmed WAXS spectra of M1 (a). Interplay among the d‐spacing, relative crystallinity, and crystallite size (b). MSD plots (c) and the WAXS spectra (d) of M1, M2, and M3. *FFV* (e), SAXS spectra with corresponding 2D images (f), Kratky plots (g), Guinier plots (h), illustration of polymer conformations and radii of gyration (i), and Porod plots (j) derived from the SAXS profiles of M1–M3.

Heating changes the structure of polymer membranes through degradation, self‐crosslinking, softening, and crystallization processes. In our study, the polymers were not degraded because the polymer membranes were heated to a maximum of 120°C, which is below the degradation temperature (≈250°C; DSC and TGA results in Figure S1). A slight peak shift between 175 and 200 °C in the DSC curves is likely due to the distinct modification treatments applied to the membranes, which subtly influenced the polymer chain configurations. The FTIR spectra of the polymer membranes were unchanged after heat treatment (Figure S2), suggesting the absence of thermal crosslinking. No pronounced crystallization and melting temperature peaks appeared in the DSC curves (Figure S1a), indicating the dominantly amorphous nature of the polymer membrane. The WAXS spectra of the polymer membranes exhibited a single broad peak (Figure 2a), indicating some degree of long‐range ordering or small crystalline portions governed by interpolymer chain organization. The peak at a q‐value of ≈1.29 Å^−1^ corresponded to a d‐spacing of ≈4.86 nm, likely representing the average interpolymer chain distance.

**FIGURE 3:**
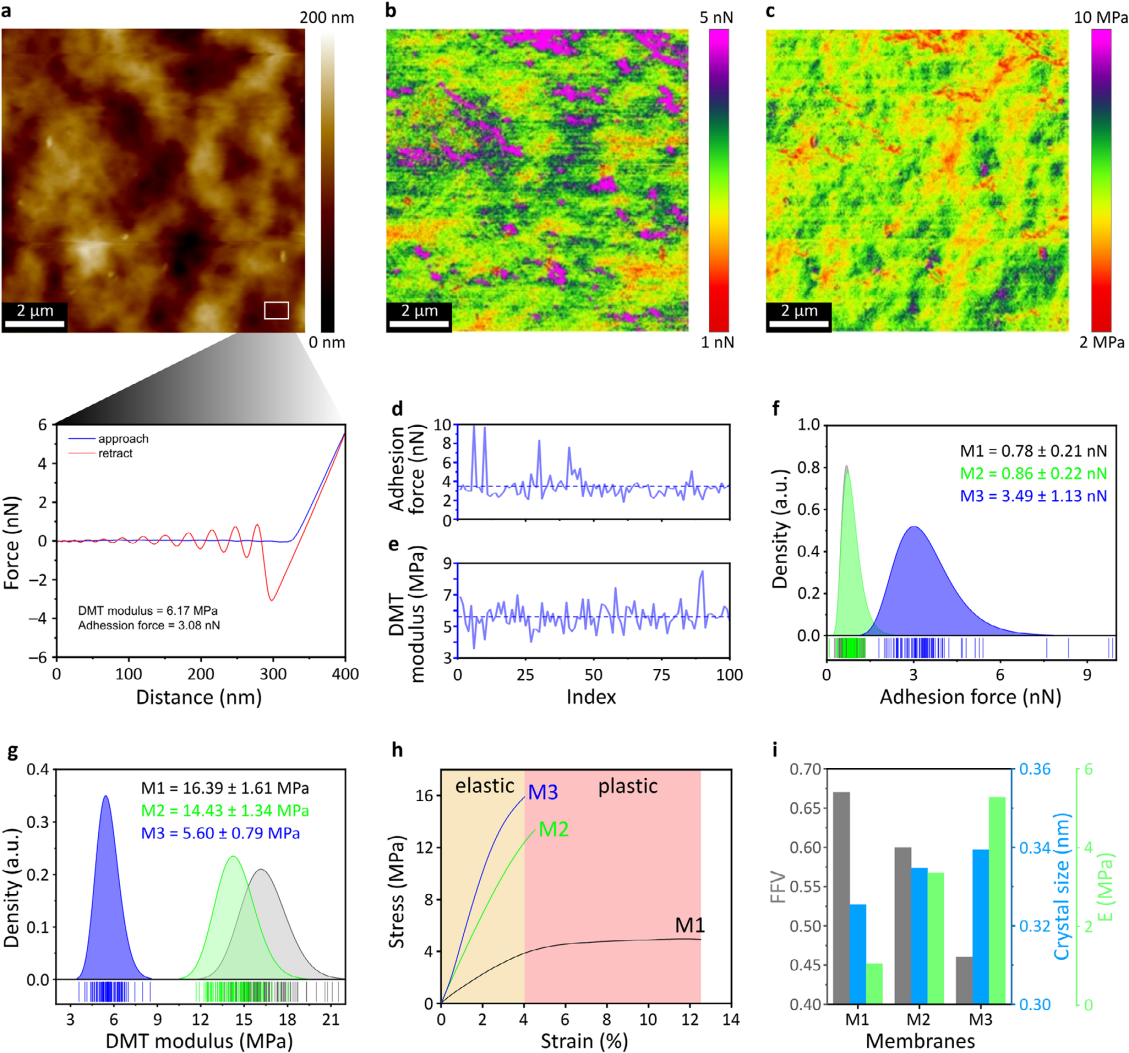
Nanomechanical analysis of the polymer membranes. AFM topographic map of M3 with force–distance characterization at each pixel (256 × 256), also showing a force–distance profile of one location on the membrane surface (a). Distribution maps of adhesion forces (b) and DMT moduli (c) of M3. Fluctuations in adhesion forces (d) and DMT moduli (e) obtained from 100 locations on the surface of M3. Distributions of adhesion forces (f) and DMT moduli (g) of M1, M2, and M3. Stress–strain curves of M1, M2, and M3, (h) and interplay between *FFV*, crystallite size, and Young's modulus (i).

The polymer softening behavior and evolution in the crystallinity were observed when the polymer membranes were exposed to various thermal treatments, as evidenced by pronounced alterations in the scattering spectra of the samples obtained via in situ temperature‐programmed WAXS (Figure 2a). As the polymer membranes were gradually heated from room temperature (25°C) to 120°C, peaks in the WAXS spectra shifted to a lower q‐value. The q‐value further reduced under sample isothermal treatment at 120°C for 2 h, implying that heating increased the d‐spacing between the polymer chains (Figure 2b). Interestingly, when the polymer membranes were cooled from 120°C to room temperature (25°C), the q‐value of the WAXS peaks shifted to a higher q‐value than the initial value. This indicates that heating followed by cooling caused an irreversible structural transition that reduced the interpolymer chain distance. The reduction in the interpolymer chain distance increases the rigidity of the polymers, which is reflected in the higher Young's moduli of M2 and M3 compared with M1, as measured by DMA (Figure 3h). This polymer chain rearrangement is also evident from the peak shift observed in the DSC curves between 175 and 200°C (Figure S1).

The peak intensity of the WAXS spectra is correlated with the crystallinity of materials. Quantification of the absolute crystallinity requires the reference of fully crystalline polymer counterpart, which did not exist in this case. Therefore, we compared the crystallinities of the studied polymers to each other under various heat treatments, i.e. obtaining their relative crystallinities. Heating increased the intensity of WAXS peaks, indicating an increase in the crystallinity of the polymer membranes. Upon cooling from 120°C to room temperature, the crystallinity slightly decreased but remained above its initial pre‐heated level. The polymer chains of some polymers aligned into more ordered structures during heat‐induced crystallization [28].

In WAXS, reflections from very small crystallites are broadly distributed in the spectrum and overlap with the spectral components of amorphous phases [29]. The sizes of crystallite portions in the fabricated polymer membranes increased with increasing temperature (Figure 2b). This may occur due to the partial melting of the smaller crystallites that fused into larger crystallites during the recrystallization process. These structural transformations were possible because the polymer chains gain enough kinetic energy to move more freely upon heating, allowing the materials to soften and reorganize the polymer chain arrangements.

To support the experimentally observed polymer softening behavior, we evaluated polymer‐chain flexibility under various temperature treatments through MD simulations. The extent of polymer chain mobility and flexibility can be represented by the time dependence of the *MSD* [30]. In this study, we averaged the *MSD*s obtained over the simulation time scale. Upon heating at 120°C, the polymer chains exhibited the highest average MSD value of 17.9 Å^2^, which was larger than that at initial room temperature (8.8 Å^2^), i.e. M1 (Figure 2c). It was inferred that raising the temperature increased the flexibility of the polymer chains. Upon cooling from 120°C to room temperature (M2), the average *MSD* value decreased to 4.4 Å^2^, lower than the initial *MSD* of the unheated membrane M1. Moreover, applying pressure during heating (M3) resulted in polymer structure with the lowest *MSD* (3.9 Å^2^), indicating the most rigid configuration. The low *MSD* values observed after heating followed by cooling indicated that the thermal treatments reduced the flexibility of the polymer chains compared to their initial flexibility.

From the in situ temperature‐programmed WAXS and MD simulation, we concluded that as the polymer chains gained kinetic energy and freedom of movement during heating, they became entangled and failed to recover their initial configuration during cooling. These conditions reduced the interpolymer chain distance and enhanced the formation of crystalline contents, thus reducing the flexibility of the polymer chains.

The WAXS spectra of M1, M2, and M3 exhibited similar patterns with a broad peak and a shoulder at q‐values of ≈1.29 Å^−1^ and ≈2.96 Å^−1^, respectively (Figure 2d). The main peak in the scattering pattern was attributed to the average interpolymer chain distance (d‐spacing), while the shoulder was assignable to the presence of a minor, less‐ordered packing component. From the experimentally measured densities (0.46, 0.58, 0.76 g cm^−3^ for M1, M2, and M3, respectively), the *FFV*s of M1, M2, and M3 were calculated as 0.67, 0.60, and 0.46, respectively (Figure 2e).

The obviously different SAXS patterns of M1–M3 (Figure 2f) indicate structural variations in local ordering. A distinct diffused rings, indicating a local arrangement consisting of stacked amorphous and crystalline layers [32], was clearly observed in the SAXS image of M1 (Figure 2f, inset). The SAXS spectrum of M3 was more profoundly different than those of M1 and M2, showing lower intensity than those of M2 and M3 (Figure 2f) and no ring formation in the 2D scattering image. These observations suggest that combined compression and heat treatments more substantially influence the local structure of the polymer membrane than heat treatment alone.

The degree of unfolding in the polymer membranes, which is correlated with polymer‐chain flexibility, was qualitatively evaluated from the Kratky plots (Figure 2g) derived from the SAXS profiles. The Kratky plots of M1, M2, and M3 all display lower y‐axis values at high q.R_g_, suggesting that the polymer chains formed a coil arrangement within a globular shape [33, 34]. Nevertheless, the plot of M1 displayed a higher plateauing tendency at higher q‐values than the plots of M2 and M3, indicating a partially unfolded (extended) conformation between the globules. Furthermore, the radius of gyration (*R*_g_), which measures the average distance of the monomers in the polymer chain within the globule from their common mass center, was quantified through a Guinier analysis (Figure 2h). The *R*_g_ increased in the order of M1 <M2 <M3, with values of 40, 45, and 55 Å, respectively. From the Kratky and Guinier analyses, we inferred that annealing and hot‐pressing followed by slow cooling induced polymer‐chain arrangement within the globule became more extended but the conformations between the globules became more compact, as illustrated in Figure 2i.

Some amorphous solid materials consist of fully random molecular arrangements forming a continuum of constant density; others contain supramolecular domains of different densities and more‐or‐less pronounced boundaries [12]. The density fluctuations in a multiphase system can be evaluated using Porod's law [35]. The theoretical Porod plots of various amorphous systems with different densities and boundary conditions are shown in Figure S3. In an ideal two‐phase system with sharp boundaries and constant but different electron densities in each phase, the Porod plot exhibits a constant value at high q‐ranges. In a quasi‐two‐phase system with a diffuse phase boundary or a transition zone (i.e., an interface layer), the scattering intensity is reduced, especially at high q‐values, and the plot negatively deviates from Porod's law. Membranes M1–M3 exhibited a positive deviation from Porod's law, indicating a quasi‐two‐phase system with sharply defined phase boundaries and fluctuating electron densities within the phases (Figure 2j).

Furthermore, the nanoscale mechanical properties of the polymer membranes were investigated through AFM quantitative nanomechanics. Figure 3a shows the AFM topographic map of M3 along with a force–distance curve generated from a single point measurement representing a lateral dimension of ≈2 nm (limited by the apex radius of the AFM tip). To map the nanomechanical properties, 256 × 256 points were measured within a 10 × 10‐µm^2^ membrane area, generating 65,536 force–distance curves. These curves were then used to determine the adhesion force and Derjaguin–Muller–Toporov (DMT) modulus of the membranes. The adhesion force, which represents the attractive force between a material surface and the AFM cantilever, was obtained by measuring the sudden deflection changes as the tip was retracted from the sample surface [36]. The DMT modulus, which is related to the stiffness or rigidity of a material surface, was determined from the force–distance curve in the retraction region.

The adhesion forces and DMT moduli were unevenly distributed across the membrane surface (Figure 3b,c). Figure 3d,e show fluctuations in the adhesion forces and DMT moduli, respectively, measured at 100 representative locations over the membrane surface. The average adhesion forces on M1, M2, and M3 were 0.78 ± 0.21, 0.86 ± 0.22, and 3.49 ± 1.13 nN, respectively. The range of adhesion forces was wider for M3 than for the other membranes, as evidenced by the broad distribution curve in Figure 3f. This suggests larger variability in surface chemistry, hydrophobicity, and tip–sample contact area heterogeneity. The average DMT moduli of M1, M2, and M3 were 16.39 ± 1.61, 14.43 ± 1.34, and 5.60 ± 0.79 MPa, respectively. Interestingly, the average adhesion forces and DMT moduli of M1 and M2 were very similar and obviously differed from those of M3, suggesting that heating at 120°C (M2) did not noticeably modify the chemical and nanomechanical properties of the membrane surface. On the contrary, hot‐pressing (M3) substantially affected the surface nanomechanical properties. The nanomechanical analyses of all the membranes were performed in a similar manner; the obtained results are provided in Supporting Information (Figures S7–S14).

On the bulk scales, the rigidity or stiffness of polymer membranes is represented by the Young's modulus (*E*). From stress–strain curves (Figure 3h), the Young's moduli of M1, M2, and M3 were determined to be 1.03, 3.35, and 5.28 MPa, respectively. The higher Young's moduli of M2 and M3 than that of M1 revealed that annealing and hot‐pressing increased bulk rigidity and stiffness. The pristine polymer membrane (M1) exhibited ductile characteristics with elastic behavior at strains of up to ≈4% after which it demonstrated appreciable transition to plastic behavior at higher strains, and breakage at 12% strain. Meanwhile, the thermally treated M2 and M3 membranes exhibited the characteristic behaviors of brittle materials: initial elastic behavior followed by abrupt breakage at ≈4% strain before plastic behavior could be observed. Moreover, hot‐pressed M3 demonstrated more brittle characteristics than annealed M2. The tensile behavior was further examined through MD simulations. The simulated stress–strain curves (Figure S16) exhibited the same overall trend as the experimental measurements (Figure 3h), with M3 showing the highest tensile strength and stiffness, followed by M2 and M1. The discrepancy in absolute stress–strain values between simulation and experiment is expected and can arise from timescale mismatch, where MD simulations deform the material at a much faster rate than experiment [37]. To further understand the chain orientation of the polymers during stretching, the spatial orientation correlation functions were calculated (Figure S17). All three membranes displayed a pronounced peak at short distances (≈1 Å), reflecting local chain alignment under tensile loading. The higher peak intensity observed for M3 suggested a greater degree of segmental orientation relative to M1 and M2, consistent with its superior mechanical performance.

**FIGURE 4:**
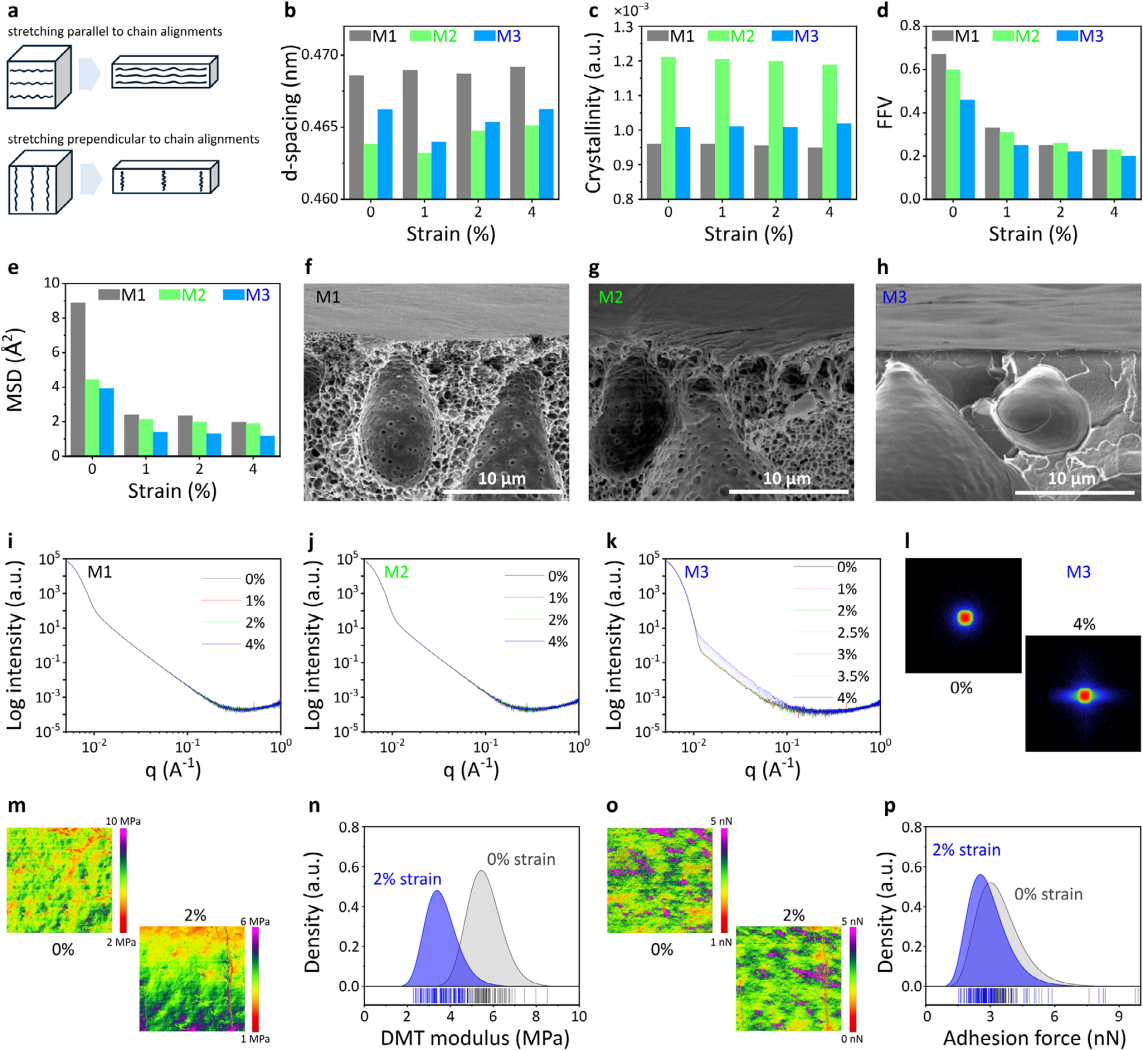
Structural evolution of M3 during uniaxial stretching. Models of chain alignments in stretched polymer membranes (a). Evolution of interpolymer chains (b), crystallinity (c), *FFV* (d), and *MSD* (e) in stretched M1, M2, and M3 membranes. Cross‐sectional scanning electron microscopy images of M1 (f), M2 (g), and M3 (h). SAXS spectra of M1 (i), M2 (j), and M3 (k) at various strains. 2D scattering images of M3 under 0% and 4% strain (l). Maps of DMT moduli over a 10 × 10‐µm^2^ area of the membrane (m) and DMT modulus distributions in M3 under 0% and 2% strains (n). Adhesion force maps over a 10 × 10‐µm^2^ area of the membrane (o) and adhesion force distributions in M3 under 0% and 2% strains (p).

Interestingly, the bulk mechanical property (Young's moduli) of the membranes showed a trend opposite to that of the surface nanomechanical property (DMT moduli). The Young's modulus increased in the order of M1 < M2 < M3, whereas the DMT modulus increased in the order of M3 < M2 < M1. As highlighted by these findings, the surface mechanical behaviors of polymer membranes do not necessarily reflect their bulk behaviors. Due to the inherently low thermal conductivity of polymer materials, a significant thermal gradient develops across their thickness during annealing and hot‐pressing. The outer surface is more directly exposed to rapid temperature fluctuations, which leads to faster heating and cooling rates compared to the inner core (bulk). This abrupt thermal exposure often prevents sufficient time for polymer chains near the surface to reorganize into an ordered crystalline structure, resulting in the formation of a less crystalline skin layer. In contrast, the inner regions (bulk) of the polymer, experiencing slower thermal transitions, allow for more effective chain rearrangement and crystallization. A shoulder peak at q ≈ 2.9 Å^−^^1^, visible only in the bulk‐sensitive WAXS spectra and absent in the surface‐sensitive GIWAXS spectra, further confirms that the bulk is more crystalline than the surface (Figure S4m,n). This skin–core structural heterogeneity has also been observed in both injection‐molded and hot‐pressed polymers [38, 39]. As a result, even though annealing and hot pressing increase overall density and crystallinity in the bulk, the surface may not reflect these changes, leading to mechanical discrepancies, especially under nanoscale probing techniques such as AFM nanomechanics. While porosity plays an important role in mechanical properties, it is not the only factor. Although M2's bulk morphology is more similar to M1 than M3, as seen in their macro‑porosity (Figure 4f–h), its bulk mechanical properties are closer to M3 (Figure 3h). This suggests that bulk mechanical behavior is influenced not only by macro‐porosity but also by molecular‐level factors (such as FFV, MSD, crystallinity, and chain folding), which are affected by thermal treatment. In contrast, surface mechanical properties are likely governed more by these molecular‐level factors because the top layer is dense.

The bulk characteristics (*FFV*s, crystallite sizes, and Young's moduli) of the polymer membranes and their correlations are shown in Figure 3i. Heating and hot‐pressing increased the crystallite size and decreased the *FFV*, reducing the amount of space for free movement of the polymer. Consequently, the polymer chains became more rigid (increasing the Young's modulus) and more prone to breakage under stress. After cooling, the annealed and hot‐pressed polymer membranes became more rigid (stiffer) and more brittle because their crystallinity increased and their conformation transformed from extended (less folded) to compact globules (more folded). The decreased *MSD* and *FFV* values confirmed the reduction of free‐movement space for the polymer after annealing and hot‐pressing, further increasing the rigidity. By correlating the WAXS–SAXS results with the mechanical analysis results and MD simulations, we elucidated the mechanical–structural relationship in the polymer membranes. Specifically, the density variations in the amorphous domains, revealed from the SAXS analysis, translated into nanomechanical inhomogeneity within the polymer membranes. We demonstrated that annealing and hot‐pressing treatments can tune the mechanical properties of polymers. Moreover, we emphasized that the surface mechanical properties of polymer membranes may substantially differ from those of bulk, necessitating independent evaluations of their contributions to the membrane performance.

### 3.2 Structural and Nanomechanical Dynamics During Uniaxial Stretching

To investigate the effects of membrane morphology on the structural dynamics during stretching, we analyzed the WAXS and SAXS spectra of M1, M2, and M3 under strains of 0%, 1%, 2%, and 4%. The WAXS spectra of M1, M2, and M3 were not obviously altered by stretching, indicating that the membranes maintained their long‐range ordering (Figure S4). Scrutinizing the WAXS profiles of the uniaxially stretched membranes, we observed a slight variation in the interpolymer chain distance (Figure 4b). The d‐spacing is expected to decrease and increase in the stretching directions parallel to and perpendicular to the polymer‐chain alignments, respectively (Figure 4a). Meanwhile, a crystallinity increase indicates that some of the coiled amorphous domains are stretched into a more aligned confirmation with a more ordered structure, whereas a crystallinity decrease implies that stretching deteriorates some of the existing crystalline regions. There was no clear trend observed in the evolution of these structural properties under uniaxial stretching (Figure 4b,c), indicating that the polymer membranes were structurally inhomogeneous.

The *FFV* (a measure of free space in polymer arrangements) and *MSD* (a measure of polymer‐chain flexibility) values decreased with increasing strain in MD simulations (Figure 4d,e), indicating that M1, M2, and M3 were densified under increasing stress. The *FFV* of M1 was the highest (0.67) and that of M3 was the lowest (0.46), suggesting that M1 and M3 were the most open and most densely packed structures, respectively. The *MSD* values decreased at high strains, highlighting restricted molecular mobility. The *MSD* of M1 was the highest (8.89 Å^2^) and that of M3 was the lowest, indicating that M1 and M3 exhibited the highest degree of segmental motion and the highest rigidity, respectively. Both M1 and M2 exhibited an integrally skinned asymmetric morphology, with a very thin layer on the top surface and large finger‐like macrovoids in the cross‐section (Figure 4f, g). These morphologies corroborated with the high *FFV* values of M1 and M2 (0.67 and 0.6, respectively), indicating that the polymer membranes could easily relax due to large empty spaces during mechanical stretching. A large FFV lowers the internal stress of a membrane, enhancing its adaptability to mechanical strain without changing its structural configuration. In M3, the finger‐like macrovoid profiles were less pronounced, and the membrane appeared to be dense without porous structures in the macrovoids (Figure 4h). These scanning electron microscopy observations supported the MD‐simulated dense configuration of M3.

Stretching barely changed the SAXS spectra of M1 and M2 (Figure 4i,j) but distinctly altered the SAXS spectrum of M3 (Figure 4k), indicating that stretching changed the structural dynamics of the M3. As the strain increased, the 2D scattering image of M3 became elongated (Figure 4l) and the SAXS intensity gradually increased at low q‐values. The equatorial streak in the 2D scattering images of M3 is attributable to the alignment of objects during polymer stretching. Such objects include internal phase boundaries, voids, and the surfaces of macroscopic entities such as polymer chains themselves [40]. The identical SAXS spectra of M1 and M2 indicate that the microstructural properties of these membranes are less affected by strain than those of M3. This may occur because of the compact packing of the polymer segments and low *FFV* in M3, which limits the polymer‐chain flexibility. The available free volume might be insufficient to accommodate the polymer‐chain relaxation under mechanical stretching, imposing a high internal stress during adaptation to the mechanical strain. Thus, structural changes upon stretching were unavoidable in the M3 membrane.

Finally, we attempted a nanomechanical evolution study of M3, which exhibited the most notable structural change under a tensile force. Unfortunately, M3 broke before the strain reached 4% during the AFM nanomechanic measurements (Figure S22). Therefore, the nanomechanical analysis data were collected only up to 2% strain. Upon stretching, the DMT moduli (Figure 4m) were unevenly distributed and their average decreased from 5.60 ± 0.80 to 3.60 ± 0.73 MPa (Figure 4n). The adhesion forces were similarly inhomogeneously distributed (Figure 4o) with an average decrease from 3.45 ± 1.32 to 2.94 ± 1.10 nN under 2% strain (Figure 4p). The integrated structural and nanomechanical analyses demonstrated that mechanical forces alter both the bulk and local‐scale (membrane surface) properties of polymer membranes. This stretching strategy can appropriately modulate the membrane properties for various applications.

## 4 Conclusions

Recognizing the complexity of polymer membranes, they can be comprehensively understood only by characterizing their mechanical and structural properties across multilength scales. Our results demonstrated a distinct disparity between the bulk and surface mechanical properties of polymer membranes, underscoring the importance of evaluating both the regions. AFM nanomechanics uncovered the nanoscale mechanical inhomogeneity within polymer membranes, which remained inaccessible to conventional techniques such as DMA. Understanding nanoscale mechanical behavior under strain is crucial because many membrane performance metrics—such as permeability, selectivity, fouling resistance, and long‐term durability—are directly influenced by how the polymer network responds at small length scales. SAXS analysis revealed a correlation between the mechanical inhomogeneity and density variations in amorphous domains. Using in situ SAXS–WAXS spectroscopy, we also investigated the local and bulk structures of polymer membranes after thermal and mechanical treatments. The kinetic energy provided by heating enhanced the free movement of polymer chains, leading to increased entanglement that prevented recovery to original configurations during cooling. This behavior reduced the interpolymer chain distance and promoted the formation of additional crystalline domains, ultimately decreasing chain flexibility. These experimental observations were supported by MD simulations, which showed that heating reduced the *MSD* (an indicator of chain flexibility). Thermal treatment also increased the crystallite size, driven by partial melting of small crystallites that subsequently fused into larger crystallites during recrystallization. This structural reorganization was accompanied by increased density and a lowered *FFV*, reducing the space for chain mobility. Consequently, polymer chains became more rigid, as reflected in their high Young's moduli, and susceptible to fracture under applied stress. Annealing and hot‐pressing, followed by slow cooling, further influenced chain organization, extending polymer‐chain conformations within globules while compacting their arrangements. By understanding the structural and mechanical evolution across multilength scales, we can rationally design polymer membranes that meet performance requirements. We demonstrate that annealing, hot‐pressing, and stretching can tailor the mechanical and structural characteristics of polymer membranes. We anticipate that our multilength‐scale (macroscopic to molecular) characterization strategies for studying structure–property relationships are generalizable to other materials such as films, 2D materials, and fibers.

## Author Contributions

R.H.: conceptualization, methodology, data curation, formal analysis, investigation, validation, visualization, writing – original draft, review & editing; H.V.: computational modeling, investigation, writing – review & editing; Y.Y.: AFM quantitative nanomechanics data curation, formal analysis, investigation, visualization; C.S.: polymer synthesis, methodology; E.Y.‐X.C.: polymer synthesis, supervision; M.L.: conceptualization, AFM quantitative nanomechanics methodology, resources, supervision; G.S.: conceptualization, resources, methodology, writing – review & editing, supervision, funding acquisition, project administration.

## Conflicts of Interest

The authors declare no conflicts of interest.

## Supporting information




**Supporting File**: advs73845‐sup‐0001‐SuppMat.pdf.

## Data Availability

The data that support the findings of this study are available in the supplementary material of this article.
