# Supplementary material for: Structural and Mechanical Dynamics of Polymer Membranes Across Multilength Scales
Source: Adv Sci (Weinh). 2026 Jan 20;13(17):e21391. doi: 10.1002/advs.202521391 (PMC13042879; doi:10.1002/advs.202521391)
Supplement: Supplementary file 1 — Supporting File: advs73845‐sup‐0001‐SuppMat.pdf. [file ADVS-13-e21391-s001.pdf]

Supporting Information (SI)

# Structural and mechanical dynamics of polymer membranes across multilength scales

Rifan Hardian[1,2], Hakkim Vovusha[1,2], Yue Yuan[3], Changxia Shi[4], Eugene Y.-X. Chen[4], Mario Lanza[3,5,6,7], Gyorgy Szekely[1,2,*]

[1] Advanced Membranes and Porous Materials Center, Physical Science and Engineering Division,
King Abdullah University of Science and Technology (KAUST), Thuwal, 23955-6900, Saudi Arabia.

[2] Chemical Engineering Program, Physical Science and Engineering Division, King Abdullah University of Science and Technology (KAUST), Thuwal, 23955-6900, Saudi Arabia

[3] Physical Science and Engineering Division, King Abdullah University of Science and Technology (KAUST), Thuwal, 23955-6900 Saudi Arabia.

[4] Department of Chemistry, Colorado State University, Fort Collins, CO 80523-1872, USA

[5] Department of Materials Science and Engineering, National University of Singapore, 9 Engineering Drive 1, Building EA, Singapore 117575, Singapore

[6] Institute for Functional Intelligent Materials, National University of Singapore, 4 Science Drive 2, Building S9, Singapore 117544, Singapore

[7] Centre for Advanced 2D Materials (CA2DM), National University of Singapore, 6 Science Drive 2, Building S14, Singapore 117546, Singapore

[*]E-mail: gyorgy.szekely@kaust.edu.sa; Web: www.SzekelyGroup.com, Tel.: +966128082769

## Contents


1. **DSC and FTIR curves** ........ 4
2. **Description of the amorphous conditions** ........ 5
3. **Details on the WAXS and SAXS analyses** ........ 6
4. **Details on the AFM nanomechanic measurements** ........ 7
5. **Additional molecular dynamics (MD) simulation** ........ 14


## List of Figures


Fig. S1. (a) DSC curves of M1–M3 showing glass transition ($T_g$) and degradation ($T_d$) temperatures. (b) TGA curves of M1–M3 showing the degradation temperatures. ........ 4

Fig. S2. FTIR spectra of M1–M3. ........ 4

Fig. S3. Density fluctuations and boundary conditions in amorphous systems. (a) Theoretical Porod's plots for various amorphous systems illustrated in I – IV. (b) Experimental Porod's plots of M1 – M3. ........ 5

Fig. S4. Structural evolution of M1, M2, and M3 upon stretching. WAXS spectra at various strain (a, e, and i). Interplay between d-spacing, crystallite size, and relative crystallinity at various strain (b, f, and j). SAXS spectra at various strain (c, g, and k). Interplay between FFV, MSD, and Rg (d, h, and i). Comparison between WAXS and GIWAXS spectra of M1 and M2 (m and n). ........ 6

Fig. S5. The setting of AFM nanomechanic instrument. ........ 7

Fig. S6. Tip calibration: deflection sensitivity calibration. ........ 8

Fig. S7. Nanomechanical mappings of 10×10 $\mu m^2$ M1 surface at strain 0%. 256×256 points were measured within a 10×10 $\mu m^2$ membrane area to generate a total of 65,536 force–distance curves. Peak force setpoint used for this map is 15 nN. The scan frequency was 0.797 Hz, with aspect ratio of 1.00, capture direction was "up", and peak force amplitude was 200 nm. The motor speed was set to 0.2 mm/min, with a gain setting of ×1, corresponding to full-scale force display (0-300 N) in the acquisition window. The initial claw distances were 33.286 mm. ........ 8

Fig. S8. The M1 membrane broke on the way to a 6% strain. ........ 9

Fig. S9. Nanomechanical mappings of 10×10 $\mu m^2$ M2 surface at strain 0%. 256×256 points were measured within a 10×10 $\mu m^2$ membrane area to generate a total of 65,536 force–distance curves. Peak force setpoint used for this map is 5 nN. The scan frequency was

0.701 Hz, with aspect ratio of 1.00, capture direction was “down”, and peak force amplitude was 200 nm. The motor speed was set to 0.2 mm/min, with a gain setting of ×1, corresponding to full-scale force display (0-300 N) in the acquisition window. The initial claw distances were 33.759 mm. ........ 10
Fig. S10. After stretched to 4% strain, M2 did not undergo elastic deformation and cannot be restored to its original state. ........ 11
Fig. S11. The M2 membrane broke on the way to a 6% strain. ........ 11
Fig. S12. Nanomechanical mappings of 10×10 $\mu m^2$ M3 surface at strain 0%. 256×256 points were measured within a 10×10 $\mu m^2$ membrane area to generate a total of 65,536 force–distance curves. Peak force setpoint used for this map is 5 nN. The scan frequency was 0.701 Hz, with aspect ratio of 1.00, capture direction was “down”, and peak force amplitude was 200 nm. The motor speed was set to 0.2 mm/min, with a gain setting of ×1, corresponding to full-scale force display (0-300 N) in the acquisition window. The initial claw distances were 33.089 mm. ........ 11
Fig. S13. Nanomechanical mappings of 10×10 $\mu m^2$ M3 surface at strain 2%. 256×256 points were measured within a 10×10 $\mu m^2$ membrane area to generate a total of 65,536 force–distance curves. Peak force setpoint used for this map is 2 nN. The scan frequency was 0.701 Hz, with aspect ratio of 1.00, capture direction was “down”, and peak force amplitude was 200 nm. The motor speed was set to 0.2 mm/min, with a gain setting of ×1, corresponding to full-scale force display (0-300 N) in the acquisition window. The initial claw distances were 33.089 mm. ........ 12
Fig. S14. The M3 membrane broke on the way to a 4% strain. ........ 13
Fig. S15. Illustration of the methodology to determine average adhesion force and DMT modulus. ........ 13
Fig. S16. Stress–strain curves of M1, M2, and M3 obtained by molecular dynamics simulation. ........ 14
Fig. S17. Spatial orientation correlation function (SOFC) for M1, M2, and M3. ........ 14

## 1. DSC and FTIR curves

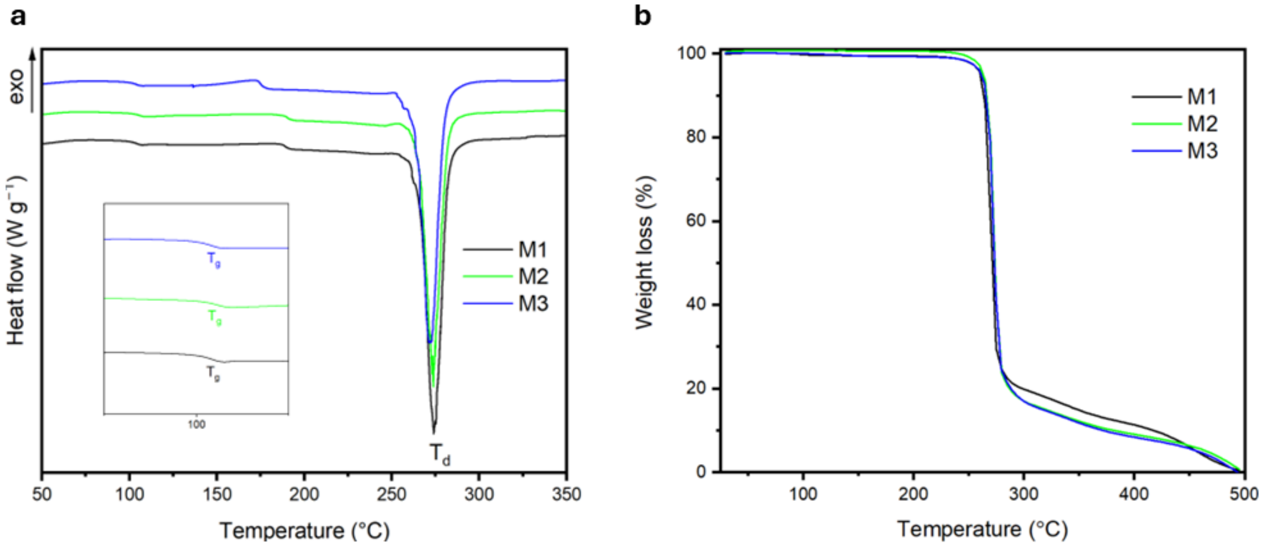


Fig. S1. (a) DSC curves of M1–M3 showing glass transition ($T_g$) and degradation ($T_d$) temperatures. (b) TGA curves of M1–M3 showing the degradation temperatures.

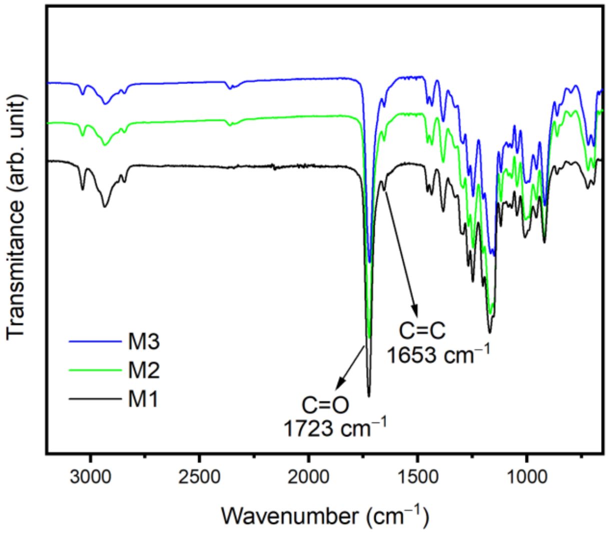


Fig. S2. FTIR spectra of M1–M3.

## 2. Description of the amorphous conditions

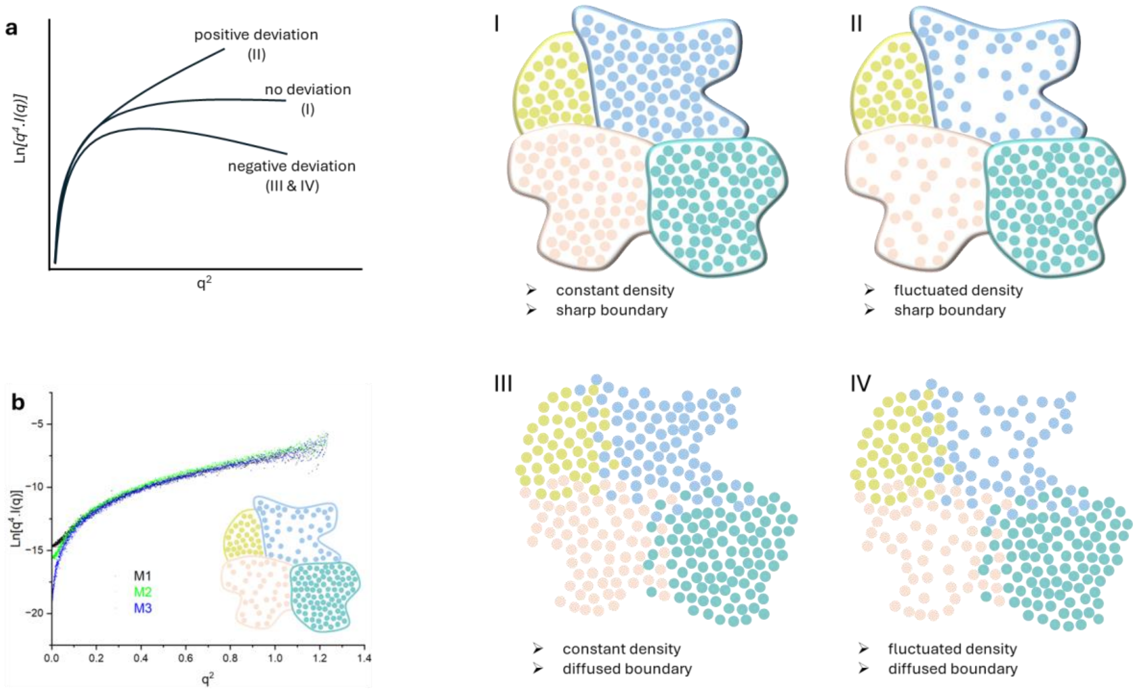


Fig. S3. Density fluctuations and boundary conditions in amorphous systems. (a) Theoretical Porod's plots for various amorphous systems illustrated in I – IV. (b) Experimental Porod's plots of M1 – M3.

## 3. Details on the WAXS and SAXS analyses

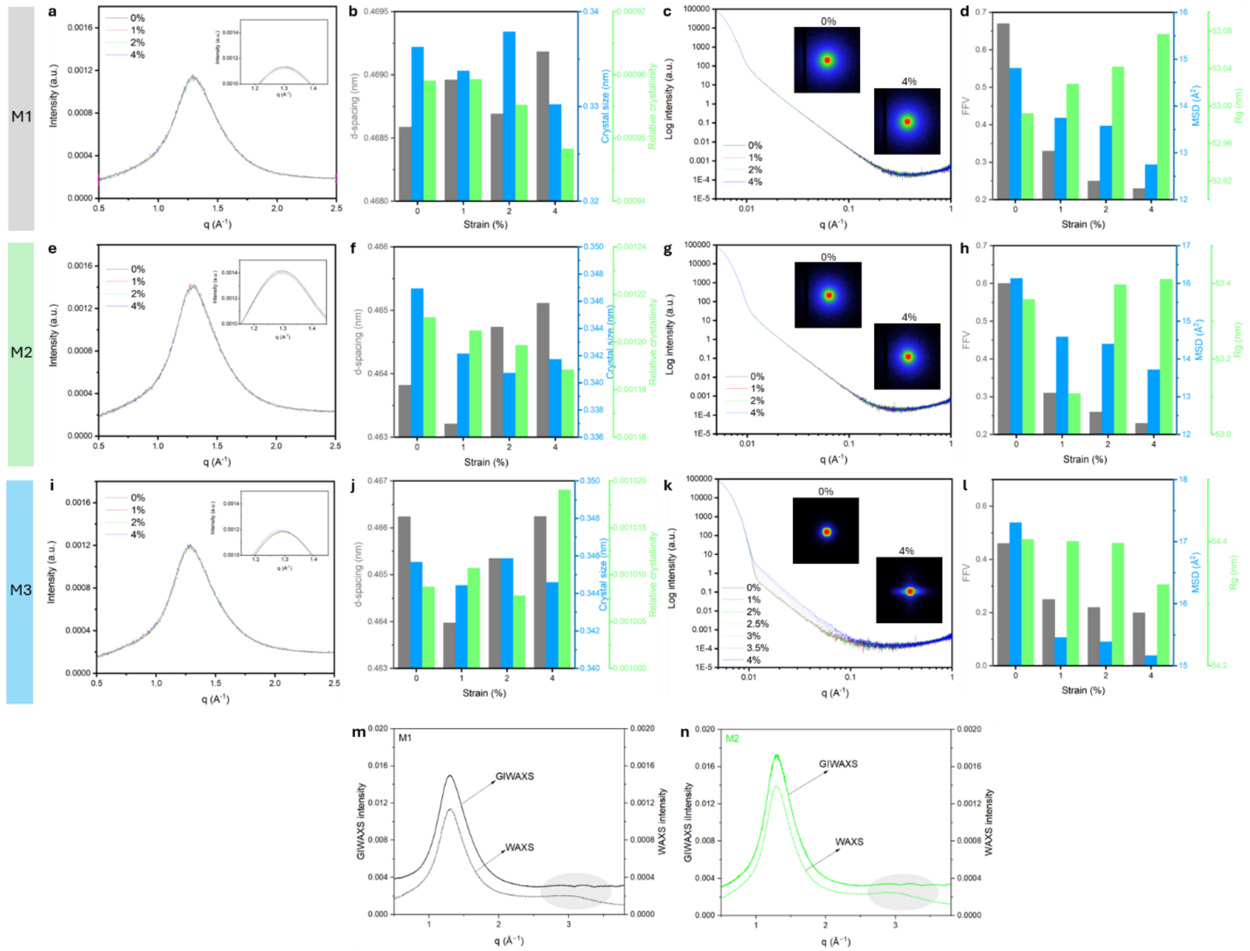


Fig. S4. Structural evolution of M1, M2, and M3 upon stretching. WAXS spectra at various strain (a, e, and i). Interplay between d-spacing, crystallite size, and relative crystallinity at various strain (b, f, and j). SAXS spectra at various strain (c, g, and k). Interplay between FFV, MSD, and Rg (d, h, and i). Comparison between WAXS and GIWAXS spectra of M1 and M2 (m and n).

## 4. Details on the AFM nanomechanic measurements

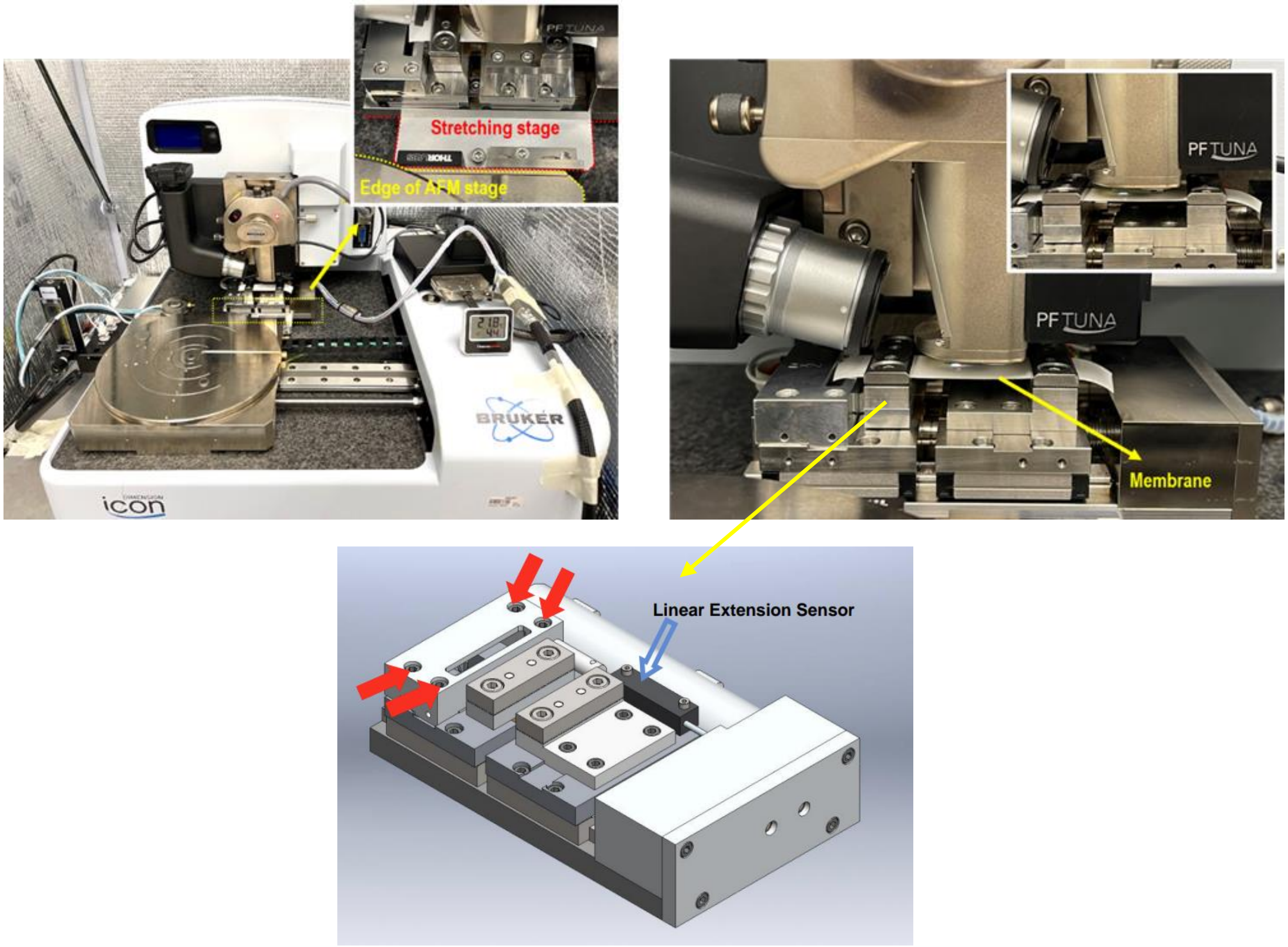


Fig. S5. The setting of AFM nanomechanic instrument.

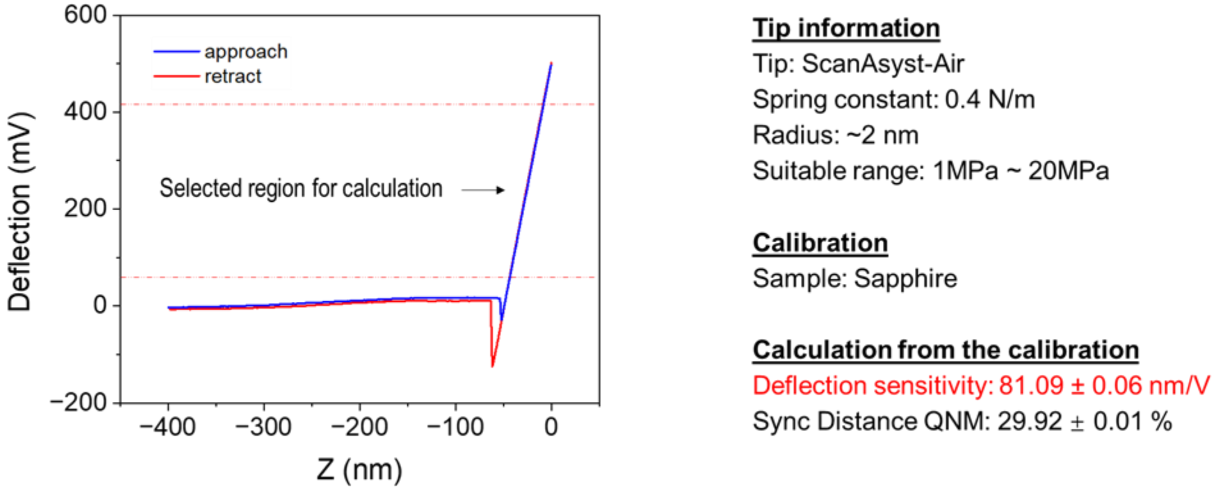


Fig. S6. Tip calibration: deflection sensitivity calibration.

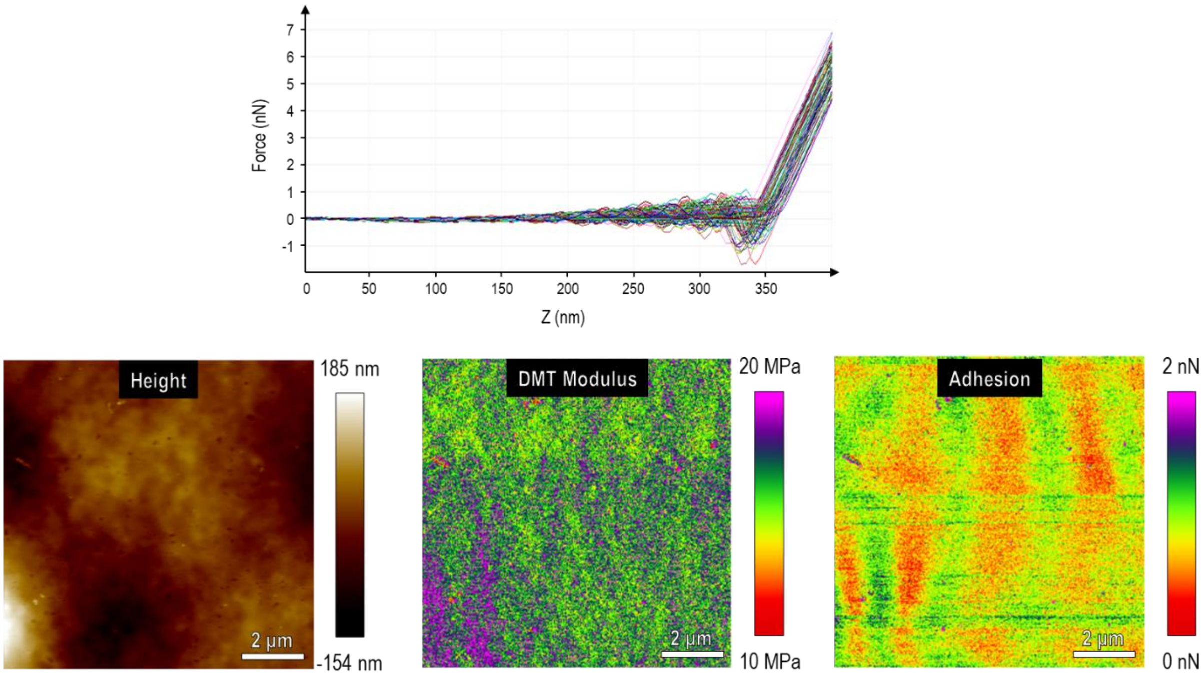


Fig. S7. Nanomechanical mappings of 10×10 μm$^2$ M1 surface at strain 0%. 256×256 points were measured within a 10×10 μm$^2$ membrane area to generate a total of 65,536 force–

distance curves. Peak force setpoint used for this map is 15 nN. The scan frequency was 0.797 Hz, with aspect ratio of 1.00, capture direction was “up”, and peak force amplitude was 200 nm. The motor speed was set to 0.2 mm $min^{-1}$, with a gain setting of ×1, corresponding to full-scale force display (0-300 N) in the acquisition window. The initial claw distances were 33.286 mm.

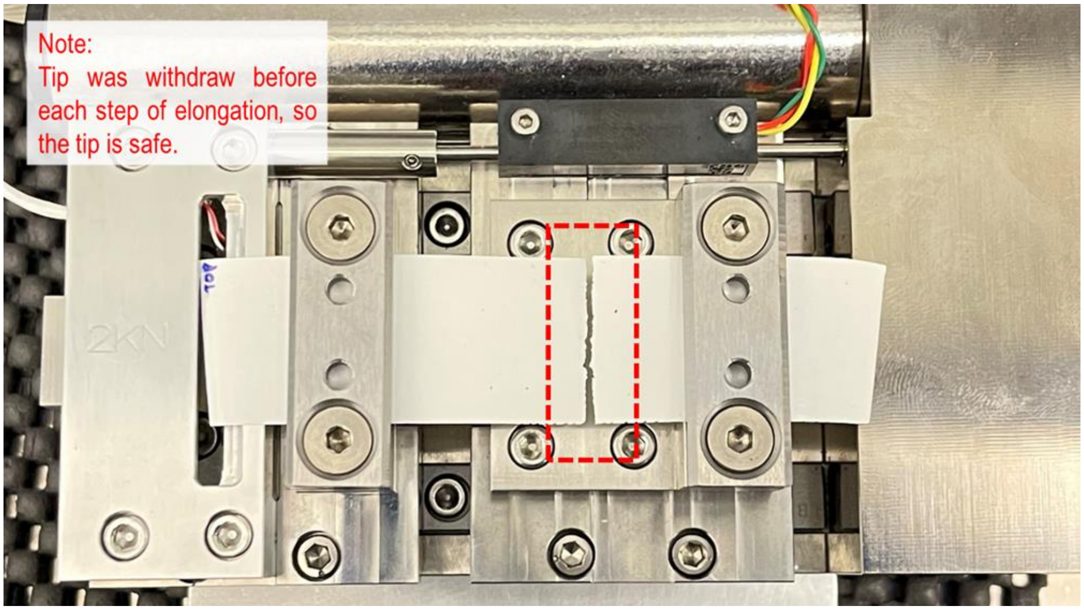


Fig. S8. The M1 membrane broke on the way to a 6% strain.

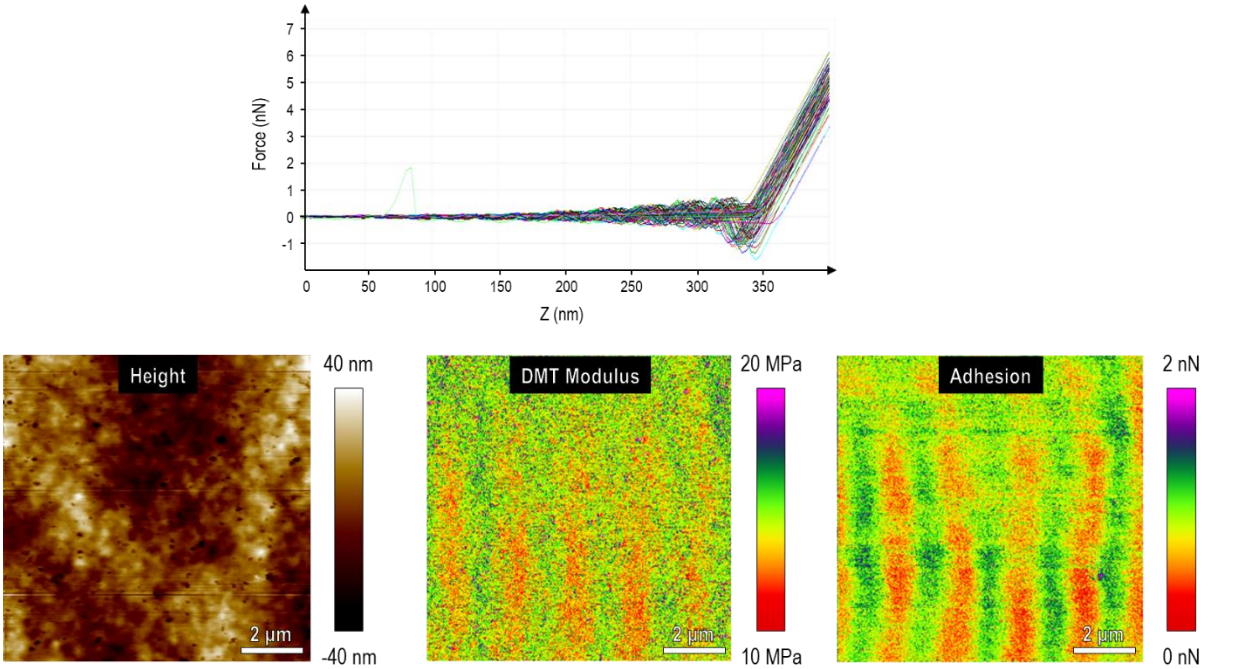


Fig. S9. Nanomechanical mappings of 10×10 μm$^2$ M2 surface at strain 0%. 256×256 points were measured within a 10×10 μm$^2$ membrane area to generate a total of 65,536 force–distance curves. Peak force setpoint used for this map is 5 nN. The scan frequency was 0.701 Hz, with aspect ratio of 1.00, capture direction was “down”, and peak force amplitude was 200 nm. The motor speed was set to 0.2 mm min$^{-1}$, with a gain setting of ×1, corresponding to full-scale force display (0-300 N) in the acquisition window. The initial claw distances were 33.759 mm.

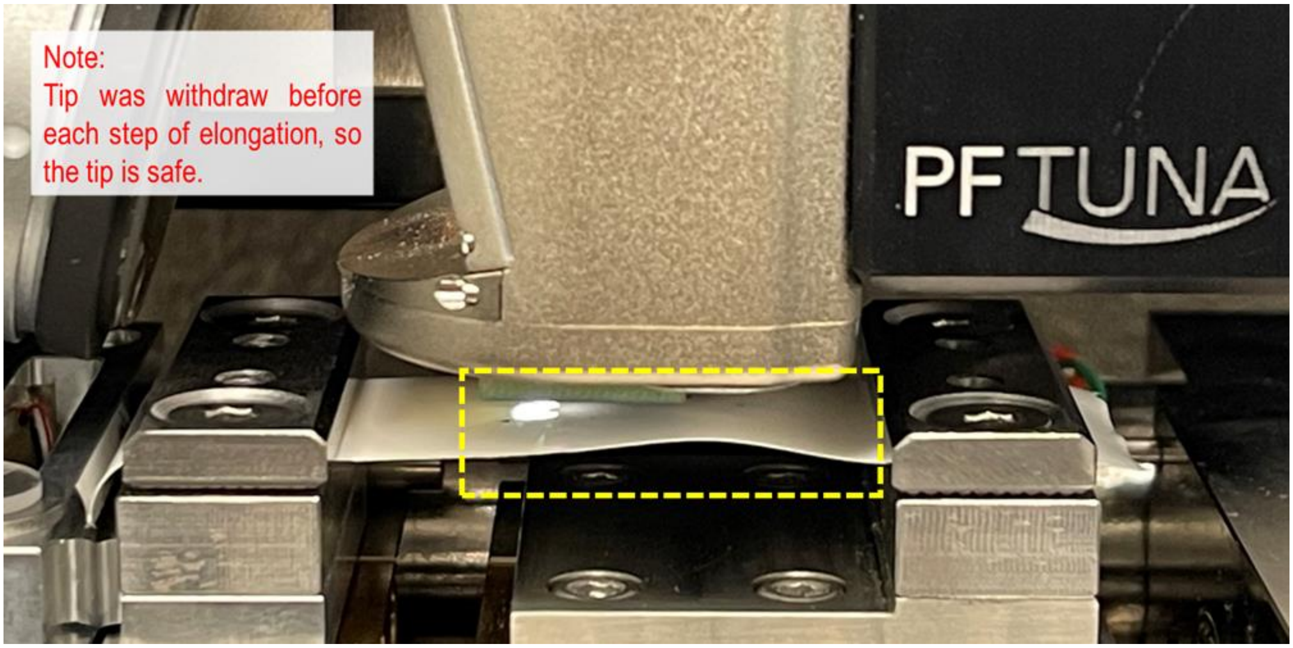

Fig. S10. After stretched to 4% strain, M2 did not undergo elastic deformation and cannot be restored to its original state.

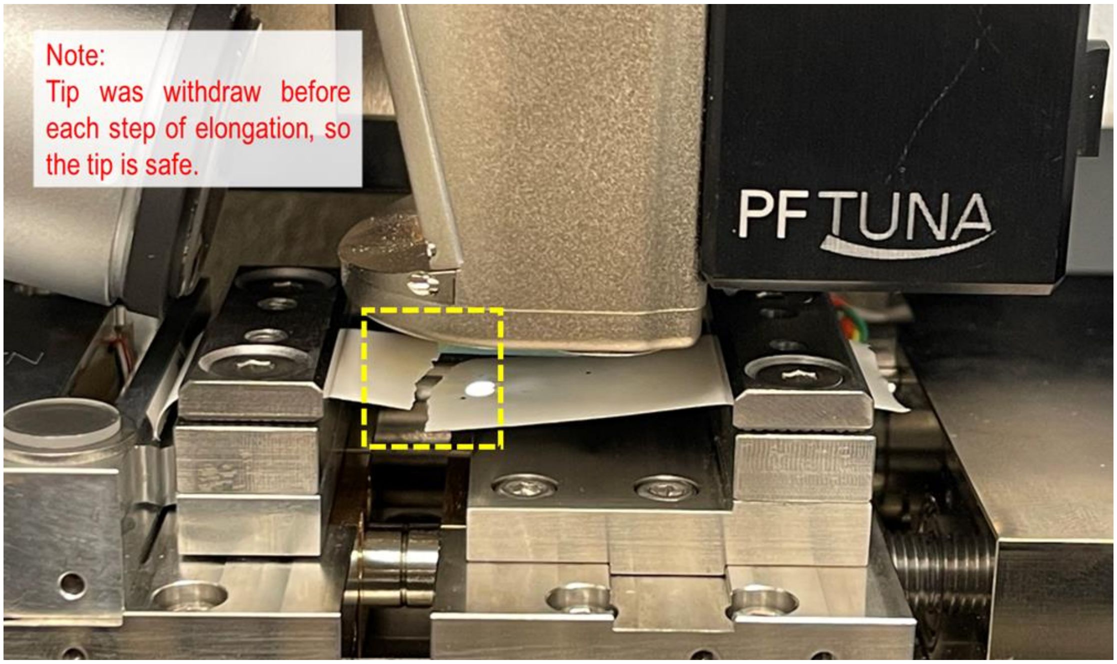


Fig. S11. The M2 membrane broke on the way to a 6% strain.

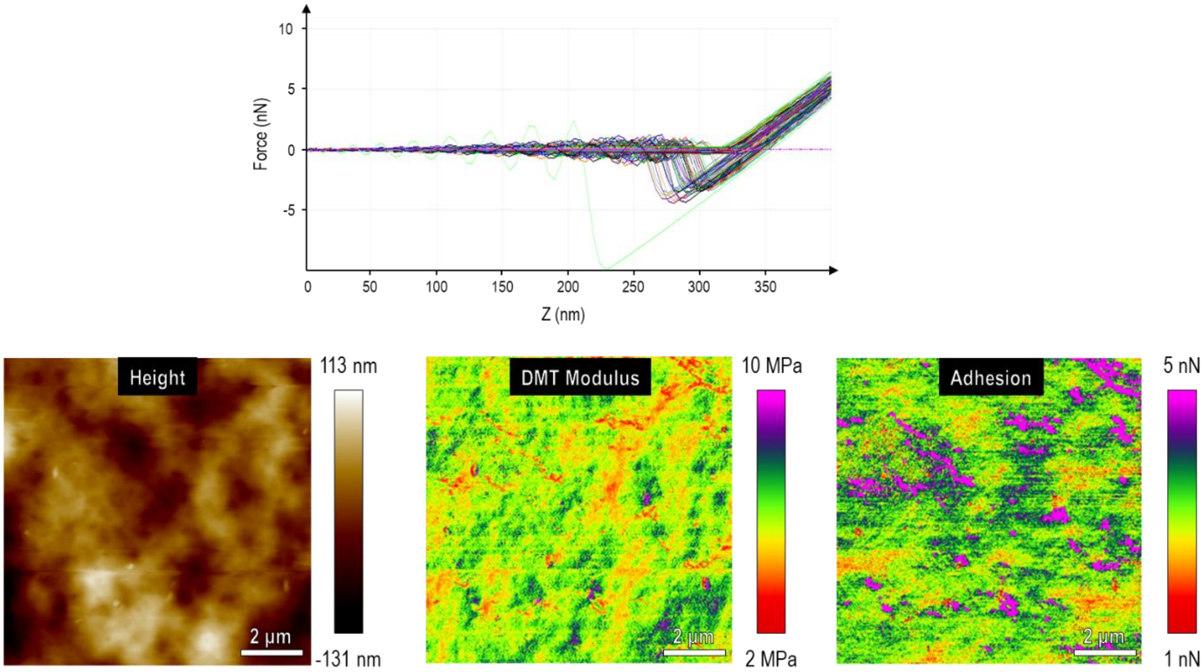


Fig. S12. Nanomechanical mappings of 10×10 μm$^2$ M3 surface at strain 0%. 256×256 points were measured within a 10×10 μm$^2$ membrane area to generate a total of 65,536 force–distance curves. Peak force setpoint used for this map is 5 nN. The scan frequency was 0.701

Hz, with aspect ratio of 1.00, capture direction was “down”, and peak force amplitude was 200 nm. The motor speed was set to 0.2 mm min$^{-1}$, with a gain setting of ×1, corresponding to full-scale force display (0-300 N) in the acquisition window. The initial claw distances were 33.089 mm.

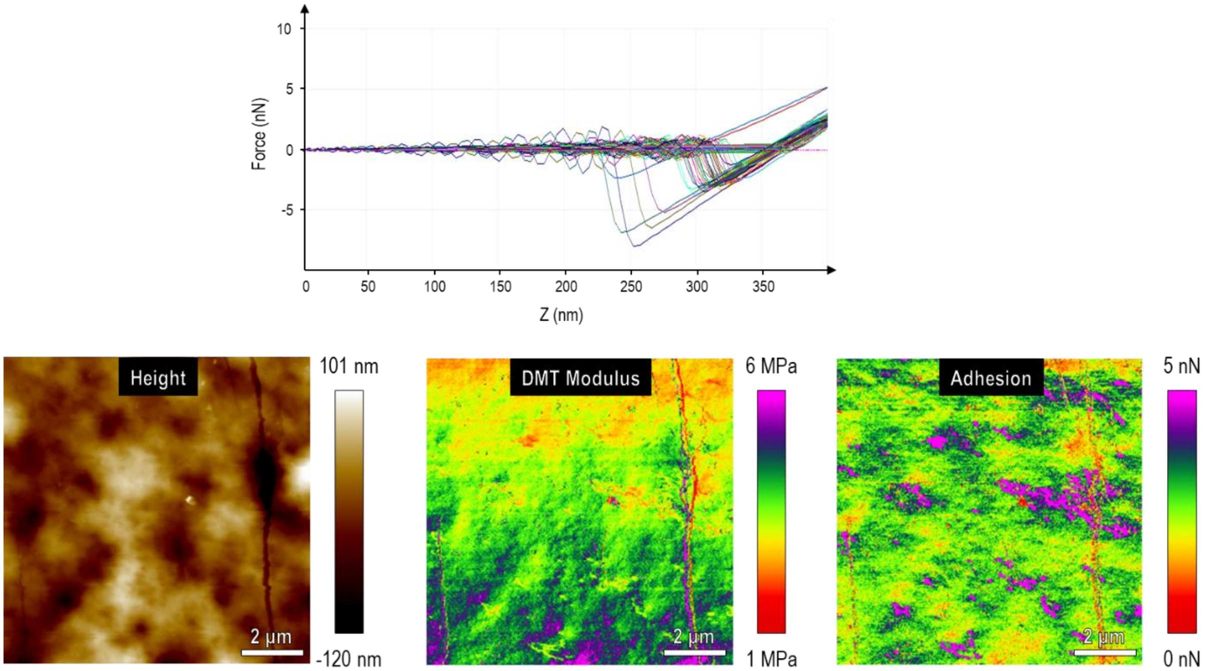


Fig. S13. Nanomechanical mappings of 10×10 μm$^2$ M3 surface at strain 2%. 256×256 points were measured within a 10×10 μm$^2$ membrane area to generate a total of 65,536 force–distance curves. Peak force setpoint used for this map is 2 nN. The scan frequency was 0.701 Hz, with aspect ratio of 1.00, capture direction was “down”, and peak force amplitude was 200 nm. The motor speed was set to 0.2 mm min$^{-1}$, with a gain setting of ×1, corresponding to full-scale force display (0-300 N) in the acquisition window. The initial claw distances were 33.089 mm.

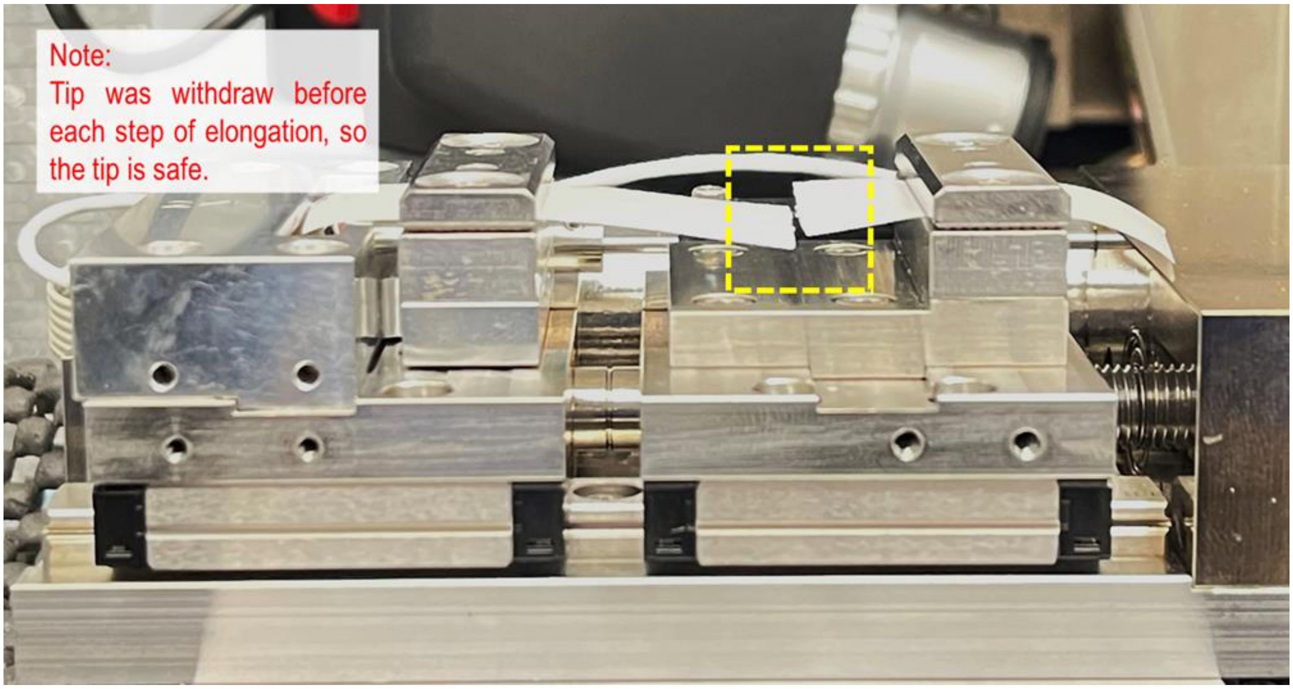


Fig. S14. The M3 membrane broke on the way to a 4% strain.

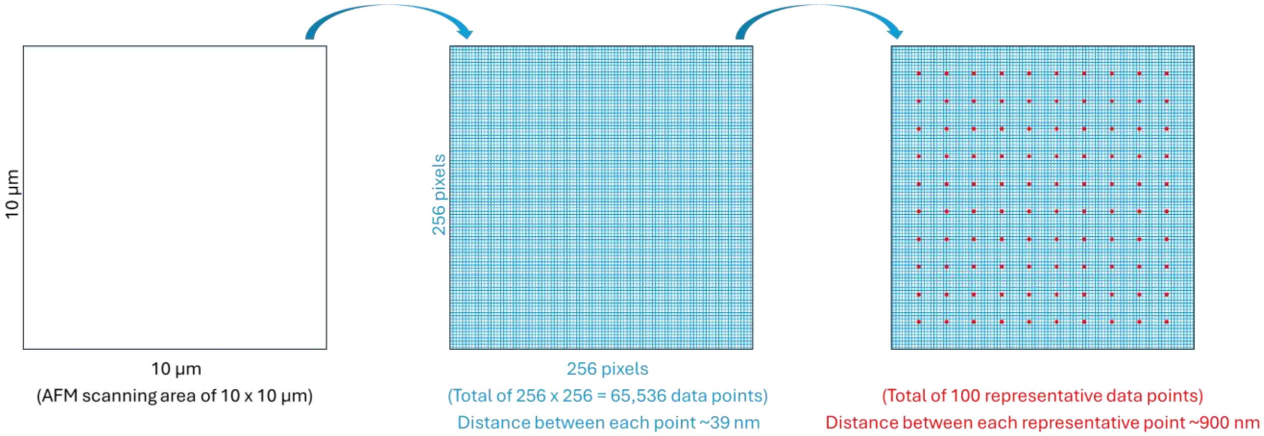


Fig. S15. Illustration of the methodology to determine average adhesion force and DMT modulus.

## 5. Additional molecular dynamics (MD) simulation

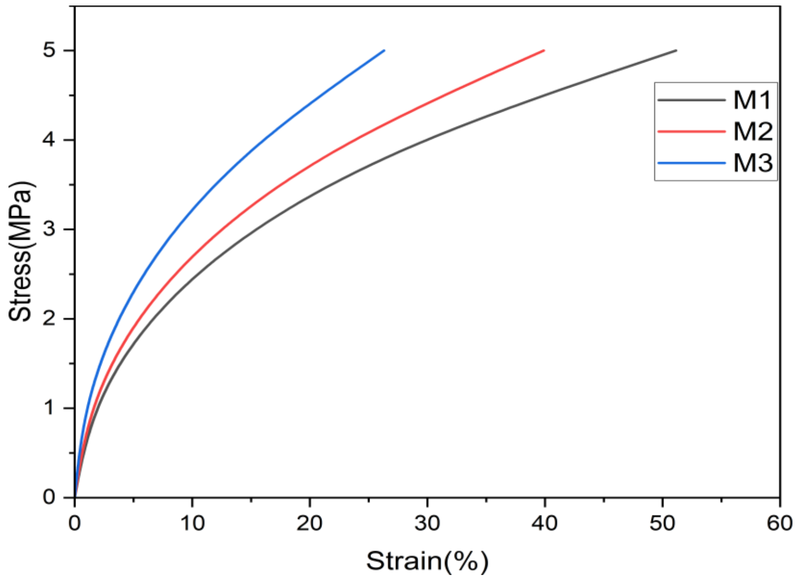


Fig. S16. Stress–strain curves of M1, M2, and M3 obtained by molecular dynamics simulation.

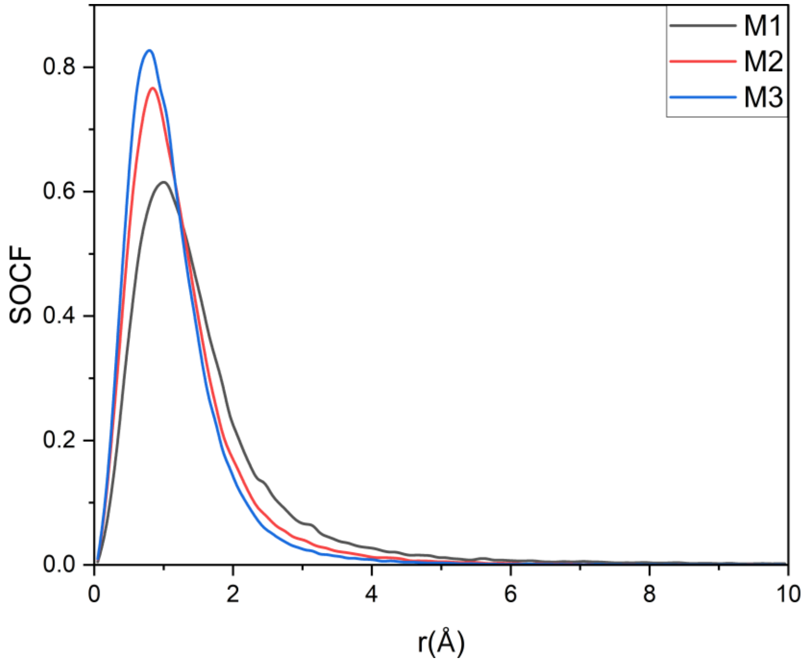


Fig. S17. Spatial orientation correlation function (SOFC) for M1, M2, and M3.
